# Metabolic control of cellular reprogramming: phospholipid remodeling tunes auxin signaling

**DOI:** 10.1093/plcell/koag239

**Published:** 2026-08-07

**Authors:** Jiajun Wang, Dandan Hu

**Affiliations:** Assistant Features Editor, The Plant Cell, American Society of Plant Biologists; School of Life Sciences, Xiamen Key Laboratory of Plant Genetics, Xiamen University, Xiamen 361102, China; School of Life Sciences, Xiamen Key Laboratory of Plant Genetics, Xiamen University, Xiamen 361102, China

Plant cells possess remarkable developmental plasticity and can regain their proliferative capacity through cellular reprogramming to form pluripotent cell masses known as callus, which serves as the foundation for plant regeneration and tissue culture. Induction of callus formation begins with explants on auxin-rich callus-inducing medium (CIM), a process tightly controlled by auxin signaling. The canonical IAA14–ARF7/19 signaling module and its downstream targets, including LATERAL ORGAN BOUNDARIES DOMAIN (LBD) transcription factors and WRKY23, are indispensable for callus formation and the establishment of cellular pluripotency (Xu et al. 2023).

Lipids are fundamental components of plant membranes that maintain membrane integrity, dynamics, and energy storage. Beyond these structural roles, increasing evidence suggests that lipid metabolism also generates signaling molecules, such as phosphatidic acid (PA), phosphoinositides, and their phosphorylated derivatives. These signaling molecules modulate protein kinase activity, membrane trafficking, cytoskeletal reorganization, and hormone signaling, thereby impacting diverse developmental and stress-responsive processes (Chang et al. 2026a). However, whether lipid metabolism participates in callus formation and subsequent plant regeneration has remained unclear. In new work, Pengjie Chang and colleagues (Chang et al. 2026b) demonstrate that phospholipid remodeling is essential for auxin-induced callus formation during Arabidopsis regeneration.

The authors profiled the lipidome of *Arabidopsis* root explants during auxin-induced callus formation and identified 256 lipid metabolites whose abundances changed after incubation in the liquid CIM. Notably, phospholipids showed a clear downward trend: 40 of the 50 differential phospholipid species, including PA, phosphatidylcholine (PC), phosphatidylethanolamine (PE), phosphatidylserine (PS), and phosphatidylglycerol (PG), decreased following incubation in liquid CIM. In contrast, although glycerolipids constituted the largest class of differential metabolites, they did not exhibit an overall tendency toward either increased or decreased accumulation. KEGG pathway enrichment analysis further revealed that these differential metabolites were predominantly enriched in the phospholipid metabolic pathway.

De novo phospholipid biosynthesis in plants begins with glycerol-3-phosphate (G3P), which is sequentially acylated by glycerol-3-phosphate acyltransferase (GPAT) and lysophosphatidic acid acyltransferase (LPAT) to produce phosphatidic acid (PA) (Fig. 1a). As the central intermediate of phospholipid metabolism, PA can be further converted through distinct metabolic branches into major glycerophospholipids, including PC, PE, PG, and phosphatidylinositol (PI) (Nakamura 2017). In a separate pathway, patatin-related phospholipase A (pPLA) hydrolyzes phospholipids to generate lysophospholipids and free fatty acids (FFAs), thereby contributing to membrane lipid remodeling and lipid homeostasis (Fig. 1a) (Ali et al. 2022).

**Figure 1:**
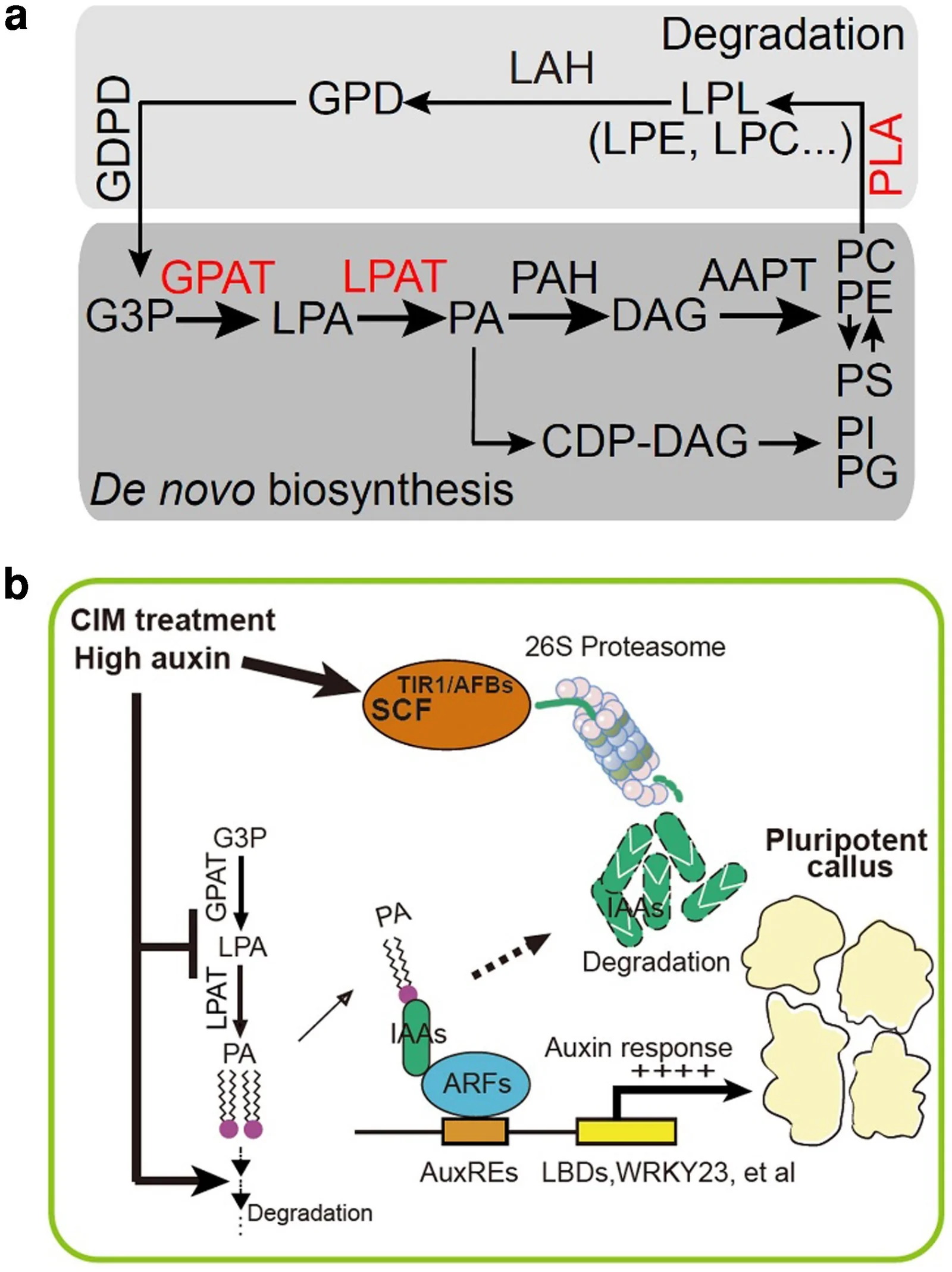
A model for phospholipid remodeling–auxin signaling crosstalk during auxin-induced pluripotent callus formation. a) Schematic overview of de novo phospholipid biosynthesis and phospholipid hydrolysis in plants. Glycerol-3-phosphate acyltransferase (GPAT) catalyzes the acylation of glycerol-3-phosphate (G3P) to produce lysophosphatidic acid (LPA), which is subsequently converted into phosphatidic acid (PA) by lysophosphatidic acid acyltransferase (LPAT). PA serves as the central intermediate for the biosynthesis of the major glycerophospholipids, including phosphatidylcholine (PC), phosphatidylethanolamine (PE), phosphatidylinositol (PI), and phosphatidylglycerol (PG). Phospholipase A (PLA) hydrolyzes glycerophospholipids to generate lysophospholipids and free fatty acids (FFAs). b) Model for the interplay between PA and auxin signaling during callus formation. On callus-inducing medium (CIM), elevated auxin levels activate the canonical SCF^TIR1/AFBs^-mediated degradation of Aux/IAA proteins while simultaneously triggering phospholipid remodeling, resulting in a decline in PA levels. PA directly binds to Aux/IAA proteins and promotes their stability. Reduced PA levels relieve PA-mediated stabilization of Aux/IAAs, thereby accelerating their degradation, enhancing auxin signaling, and inducing the expression of downstream regulators, including LBDs and WRKY23, to promote pluripotent callus formation. Reprinted from Chang et al. (2026b), Figs. 2b and 8.

The authors found that during callus induction from root explants, the decline in PA, PE, PC, PS, and PG was accompanied by reduced transcript levels of phospholipid biosynthetic genes, including *GPAT3/4/5/6/8* and *LPAT3/4*, whereas the expression of phospholipid hydrolases, including *pPLAIIα/β* and *pPLAIIIα/β*, was markedly increased. To investigate whether the phospholipid metabolic pathway is involved, the authors examined callus formation in the phospholipid biosynthetic mutants *gpat4 gpat8* and *lpat4 lpat5*, as well as in explants treated with PACOCF3, a phospholipase A (PLA) inhibitor that suppresses phospholipid hydrolysis. Both *gpat4 gpat8* and *lpat4 lpat5* mutants exhibited enhanced callus formation from primary root explants on CIM. Furthermore, *gpat4 gpat8* mutants also displayed enhanced callus formation from cotyledons, hypocotyls, and mesophyll protoplasts. In contrast, inhibition of phospholipid hydrolysis by PACOCF3 significantly impaired auxin-induced callus formation. These results indicate that reducing phospholipid biosynthesis promotes auxin-induced callus formation, whereas blocking phospholipid hydrolysis suppresses it.

To further identify the specific phospholipid species affecting callus formation, the authors compared the lipid profiles of wild-type (WT) and *gpat4 gpat8* seedlings and found PA and PS levels significantly decreased in the mutants. Supplementation of CIM with different phospholipids or FFAs revealed that only exogenous PA significantly inhibited callus formation from both WT and *gpat4 gpat8* root explants. Consistent with this finding, PA also suppressed callus formation from mesophyll protoplasts and reduced CIM-induced expression of *CYCB1;1*, a G2-to-M transition marker, in both WT and *gpat4 gpat8*.

Given the central role of auxin in callus formation, the authors next examined whether auxin levels and responsiveness were altered in *gpat4 gpat8* seedlings and how PA affects auxin signaling. Endogenous auxin levels were comparable between WT and *gpat4 gpat8* seedlings. However, the mutant produced significantly more lateral roots and exhibited stronger *DR5*-driven GFP signals, accompanied by increased *CYCB1;1* expression, indicating enhanced auxin responsiveness. Exogenous PA markedly suppressed auxin-induced lateral root formation, reduced *DR5*-driven GFP signals, and inhibited the expression of auxin-responsive genes in both WT and *gpat4 gpat8*. Notably, *gpat4 gpat8* seedlings displayed enhanced sensitivity to exogenous auxin. Together, these findings demonstrate that PA attenuates auxin responsiveness and consequently suppresses the auxin-induced callus formation program.

Using lipid–protein blotting, liposome-binding assays, surface plasmon resonance, and lipid–protein pull-down assays, the authors demonstrated that PA directly binds IAA8 and IAA14 through the conserved KR/RK motif in IAA14 (Fig. 1b). PA binding stabilized IAA8 and IAA14 by inhibiting their auxin-induced degradation. Consistent with this, the *gpat4 gpat8* mutant exhibited reduced nuclear PA and lower IAA8-GFP and IAA14-GFP protein levels, both of which were restored by exogenous PA. Finally, the gain-of-function IAA14 mutant *slr-1* suppressed the enhanced callus formation phenotype of *gpat4 gpat8*, providing genetic evidence that IAA14 acts downstream of GPAT4/8 during auxin-induced callus formation.

In summary, the authors reveal that phospholipid metabolism is dynamically remodeled during CIM-induced pluripotent callus formation, accompanied by a decline in multiple phospholipids, including PA, PC, PE, PG, and PS. Genetic and pharmacological analyses demonstrate that phospholipid remodeling plays a critical role in regulating auxin-induced callus formation. Mechanistically, PA directly binds to Aux/IAA proteins, stabilizes their protein levels, attenuates auxin signaling, and thereby suppresses auxin-induced callus formation and plant regeneration (Fig. 1b). This work uncovers a previously unrecognized link between phospholipid remodeling and auxin signaling, providing new insights into the lipid-mediated regulation of plant regeneration.

## Recent related articles in *The Plant Cell*

- Li et al. (2024) showed that auxin-activated NPC3 and NPC4 promote PA production to regulate PIN2 trafficking, thereby maintaining auxin gradients required for root growth and tropic responses in *Arabidopsis*.
- Wang et al. (2025) demonstrated that phosphatidic acid (PA) promotes the interaction between MPK3/6 and STOP1, facilitating MPK3/6-mediated phosphorylation and nuclear stabilization of STOP1 to enhance hypoxia tolerance in *Arabidopsis*. An accompanying *In Brief* by Wang and Wang (2025) highlighted the discovery that hypoxia-induced PA promotes MPK3/6-mediated phosphorylation and stabilization of STOP1.
